# *De novo* hybrid assembly of the rubber tree genome reveals evidence of paleotetraploidy in *Hevea* species

**DOI:** 10.1038/srep41457

**Published:** 2017-02-02

**Authors:** Wirulda Pootakham, Chutima Sonthirod, Chaiwat Naktang, Panthita Ruang-Areerate, Thippawan Yoocha, Duangjai Sangsrakru, Kanikar Theerawattanasuk, Ratchanee Rattanawong, Napawan Lekawipat, Sithichoke Tangphatsornruang

**Affiliations:** 1 National Center for Genetic Engineering and Biotechnology (BIOTEC), National Science and Technology Development Agency, Pathum Thani, Thailand; 2 Rubber Authority of Thailand, Bang Khun Non, Bangkok, Thailand

## Abstract

Para rubber tree (*Hevea brasiliensis*) is an important economic species as it is the sole commercial producer of high-quality natural rubber. Here, we report a *de novo* hybrid assembly of BPM24 accession, which exhibits resistance to major fungal pathogens in Southeast Asia. Deep-coverage 454/Illumina short-read and Pacific Biosciences (PacBio) long-read sequence data were acquired to generate a preliminary draft, which was subsequently scaffolded using a long-range “Chicago” technique to obtain a final assembly of 1.26 Gb (N50 = 96.8 kb). The assembled genome contains 69.2% repetitive sequences and has a GC content of 34.31%. Using a high-density SNP-based genetic map, we were able to anchor 28.9% of the genome assembly (363 Mb) associated with over two thirds of the predicted protein-coding genes into rubber tree’s 18 linkage groups. These genetically anchored sequences allowed comparative analyses of the intragenomic homeologous synteny, providing the first concrete evidence to demonstrate the presence of paleotetraploidy in *Hevea* species. Additionally, the degree of macrosynteny conservation observed between rubber tree and cassava strongly supports the hypothesis that the paleotetraploidization event took place prior to the divergence of the *Hevea* and *Manihot* species.

*Hevea brasiliensis* (Willd.) Müll.-Arg. (the Para rubber tree) is a deciduous perennial tree crop indigenous to the rain forests of the Amazonian basin in South America. It is a monoecious, outcrossing species belonging to the family Euphorbiaceae and has a chromosome number of 2*n* = 2*x* = 36. Rubber tree produces high-quality isoprenoid polymers (*cis*-1,4-polyisoprene) with unique physical properties such as elasticity, resilience and efficient heat dispersion that are unsurpassed by any petroleum-based synthetic substitutes^1^. Of the 2500 latex-producing plant species, *H. brasiliensis* is the only species that produces commercially viable quantities of high-quality natural rubber, accounting for more than 98% of total production worldwide^2^,^3^. Southeast Asian countries currently dominate rubber production and trade, accounting for more than 76% of the 11.96 tons produced globally, with Thailand and Indonesia being the two largest producers and exporters of natural rubber (http://faostat3.fao.org/; Accessed September 14, 2016). Despite originating from the Amazonian basin, rubber production in South America represents merely 2% of the total production worldwide due to the devastating spread of South American leaf blight (SALB) disease caused by ascomycete *Microcyclus ulei* in 1930s^4^. The infestation resulted in the collapse of most rubber tree plantations and the cessation of the commercial scale rubber production in Brazil and other South American countries^5^. Even though *Hevea* cultivation in Southeast Asia has not been affected by SALB, other native pathogenic fungi are still major threats to rubber production in this area. A number of commercially cultivated, high-yielding clones are susceptible to abnormal leaf fall disease caused by various *Phytophthora* species and up to 40% loss in yield has been observed upon infection of highly susceptible clones^6^. Another widespread pathogen, *Corynespora cassiicola*, is a necrotrophic fungus that causes Corynespora leaf fall disease, resulting in significant losses in natural rubber yield^7^.

In the past two decades, plant breeders have used conventional approaches of recurrent selections to obtain clones with improved latex yield and resistance to fungal pathogens^8^. However, traditional breeding is inherently time-consuming owing to long selection cycle in this species. The ability to select for commercially valuable traits at an early stage will have a tremendous impact on reducing time and resources needed to develop superior clones. The advent of DNA-based genetic markers provided an alternative strategy that allowed plant breeders to accelerate the process of cultivar development. Recent advances in sequencing technology have simplified and accelerated the discovery of sequence variants, enabling the shift from anonymous markers such as microsatellites to more ubiquitous single nucleotide polymorphisms (SNPs)^9^,^10^. The availability of SNP markers facilitates the construction of high-density genetic linkage maps, which are instrumental for quantitative trait loci (QTL) analyses and association studies with valuable agronomic traits^11^.

Several genomic resources for *H. brasiliensis* have been developed recently, including transcriptome sequencing of various tissues and the sequencing of the commercially cultivated RRIM600 genome^10^,^12^,^13^. While the first draft of the genome sequence^13^ provided an excellent source of genomic information, the assembly is highly fragmented, containing over a million contigs with an N50 length of 2.9 kb. Assembly of a complex genome is challenging in part owing to the presence of highly repetitive DNA sequences, which introduce ambiguity during the genome reconstruction. It has been estimated that repetitive DNA accounts for 78% of the rubber tree genome^13^, posing a major difficulty in the *de novo* assembly particularly when it is built exclusively from short-read data.

Although it is possible to construct a high-quality assembly for plant species with large genomes through hierarchical shotgun sequencing, the utilization of a sequencing platform capable of generating long reads that can span regions of complex repeats is likely to be more cost-effective and less labour-intensive/time-consuming. Recent development and applications of long-read sequencing technologies have shown substantial improvement in genome assemblies, primarily by joining contigs and scaffolds and spanning gaps around repetitive regions^14^,^15^. The Pacific Biosciences (PacBio) sequencing technology offers kilobase-sized reads without GC-bias or systematic errors; however, the raw data generated from this system is inherently error-prone, with an average accuracy of ~82%^16^. Availability of hybrid assembly software allows biologists to combine PacBio long-read data with complementary, high-accuracy short-read data from other platforms^17^. Nowak *et al*.^18^ demonstrated that the incorporation of additional 8 × coverage of PacBio data to the existing short-read-only assembly of the *Primula veris* genome resulted in 21% of the gaps being closed and 38% of the ambiguous positions in the gaps being filled, in addition to the 40% improvement in N50 contig size^18^. Similarly, a set of 16.5 × PacBio long reads was used to fill in 68% of the gaps existed in the Illumina-only assembly of the African cichlid^14^.

Recently, high-throughput short-read sequencing has been employed to explore DNA linkage up to several hundred kilobases in proximity ligation libraries constructed from *in vitro* reconstituted chromatin^19^. The proprietary *in vitro* chromatin conformation capture-based long-range genome assembly method called “Chicago” developed by Dovetail Genomics has been used in combination with a standard whole genome sequencing approach to generate a *de novo* human genome assembly with comparable accuracy and contiguity to the assemblies built using more expensive methods. The Chicago long-range scaffolding technique has also been utilized to improve the existing genome assemblies in the American alligator^19^, African clawed frog^20^ and cassava^21^.

Moving beyond fragmented genome assembly requires a high-quality and high-density linkage map that can help order and orient *de novo* assembled scaffolds into pseudo-chromosome scale sequences^22^. Complementary information in a *de novo* shotgun assembly and a genetic map can be integrated to generate a high-quality reference sequence. High-density linkage maps have successfully been employed to anchor scaffolds from whole genome shotgun assemblies in plant species ranging from a diploid common bean^23^ to a hexaploid bread wheat^24^. Integration of a genetic map and a genome assembly allows us to investigate ancient and recent whole genome duplications and perform comparative genomics to study genome architecture and genome evolution across species^25^.

Here, we employed complementary technologies to generate deep-coverage 454/Illumina short reads and medium-coverage PacBio long-read data for a *de novo* hybrid assembly of the *H. brasiliensis* genome from BPM24 (Bank Pertanian Malaysia 24) clone. We subsequently applied a long-range Chicago assembly technique^19^ to scaffold our preliminary assembly to obtain 1.26 Gb of assembled rubber tree sequences. BPM24 accession exhibits a high degree of resistance to two fungal pathogens, *Phytophthora* and *Corynespora*, commonly found in Southeast Asia^26^, and it is currently being exploited as genetic sources of fungal resistance in rubber tree breeding programs in Thailand. The availability of the high-density consensus linkage map constructed from two populations derived from BPM24^11^ allowed us to anchor and orient a large number of scaffolds in this new assembly. These genetically anchored sequences provided the first concrete evidence to demonstrate the presence of paleotetraploidy in rubber tree and enabled us to perform comparative analyses among the Euphorbiaceae.

## Results and Discussion

### Whole genome sequencing and assembly

BPM24 accession was selected for whole genome shotgun sequencing as it exhibits a high degree of resistance to two major fungal pathogens (Phytophthora and Corynespora) found throughout Southeast Asia. Several molecular and genetic resources, including a large number of SNP markers and two linkage maps^11^ have also been developed in this accession. Comprehensive shotgun sequences of the BPM24 rubber tree were obtained from ten libraries with insert sizes ranging from 350 bp to 12 kb, using three sequencing platforms (Roche 454 GS FLX+ and Illumina HiSeq 2000 to produce short sequencing reads, and PacBio RSII to produce single-molecule long reads; Supplementary Table S1). Ten plates of 454 GS FLX+ runs on four independent 1000-bp libraries generated 10,925,962 raw reads, totalling 6,024,553,332 bp with a mean read length of 550 bp. An 8-kb and 20-kb mate-pair library was run on a single 454 GS FLX+ plate each to produce 3,312,137 raw reads and a total yield of 1,107,168,348 bases. A single paired-end library (2 × 100 bp) was run on HiSeq 2000 to generate 785,539,628 raw reads totalling 79,339,502,428 bp. Sequencing of three PacBio libraries (insert sizes between 10–12 kb) using the latest P6-C4 chemistry on 90 SMRT cells produced 6,434,943 raw reads for a total of 56,308,060,886 bp. The average length for PacBio raw reads was 8,750 bp, and the maximum read length was 51,677 bp. Sequence data from all platforms represented approximately 68× coverage of the rubber tree genome (Supplementary Table S1). Initially, Illumina paired-end reads were assembled into contigs using SOAPdenovo^27^ and clustered with CD-HIT^28^. Cleaned 454 reads were subsequently assembled together with Illumina consensus contigs using Newbler software (Roche Applied Science, Indianapolis, USA). In order to avoid the assembly failure caused by long input FASTA sequences, the Illumina draft assemblies were first processed into 1.5 kb pseudo-reads with 300 bp overlap. The total size of 989,097 assembled contigs derived from 454 and Illumina data was 868 Mb, with an N50 contig length of 1,316 bp (L50 of 161,440 sequences; Table 1).

Repetitive sequences pose one of the biggest challenges for *de novo* genome assembly and much effort has been devoted to resolving ambiguous regions caused by repeats. The long-read PacBio platform greatly simplifies the assembly process as repetitive or otherwise ambiguous regions of a genome are, in many cases, traversed in single reads. Because of its speed and ability to perform the assembly using less than 30× coverage of long PacBio reads, we adopted a recent strategy of long read assembly-based mapping on the *De Bruijn* assembled contigs implemented in DBG2OLC^29^ to integrate the short-read contigs and PacBio sequence data. With the addition of PacBio reads, the contigs were further assembled to 1.25-Gb scaffolds, the N50 length of which increased almost 40 folds to 51,412 bp (L50 of 4,695 sequences; Table 1). The N50 length of our PacBio-scaffolded assembly was comparable to that of the preliminary Reyan7-37-97 assembly (55 kb) obtained from 193× coverage of Illumina shotgun and mate-pair data^30^.

**Figure 1:**
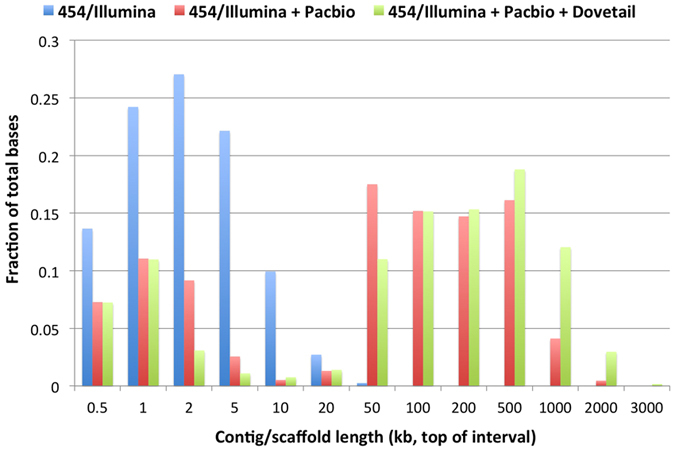
Comparison of contig/scaffold sizes at different stages of the assembly. The contig length distribution for the hybrid assembly is approximately two orders of magnitude larger compared with the short-read assembly (454/Illumina).

A combination of long-read assembly and long-range scaffolding technique offers an efficient and affordable alternative to produce a high quality reference assembly. A total of six long-range linkage libraries were constructed using proximity ligation of *in vitro* reconstituted chromatin^19^. The libraries were sequenced on an Illumina HiSeq 3000/4000 to produce 769 million 2 × 150 bp read pairs of DNA fragments between 1 to 50 kb, which provided an estimated physical coverage of 44× (Supplementary Fig. S1). Compared with the preliminary draft, the contiguity of the final HiRise assembly has almost doubled (N50 length 51.4 kb vs. 96.8 kb; Fig. 1), and the maximum scaffold length has increased from 1.22 Mb to 2.02 Mb (Table 1). Our BPM24 genome assembly spans a total length of 1.26 Gb and consists of 47,154 scaffolds larger than 1 kb (Table 1). This assembly is slightly larger than the 1.1 Gb RRIM600 draft assembly reported by the Rahman *et al*.^13^, but smaller than the 1.37 Gb assembly of Reyan7-37-97 accession recently published by Tang *et al*.^30^. The GC content of the assembled genome is 34.31%, comparable to the numbers previously reported for rubber tree^13^,^30^.

For large and complex plant genomes, low-cost short-read sequence data, even at high coverage, are hardly sufficient to obtain a highly contiguous reference-quality assembly. This is due primarily to repetitive DNA content on both large and small scales, which can occupy up to 80% of the genome in some species^31^,^32^. In addition to retrotransposons and interspersed nuclear elements, those difficult-to-assemble regions include large paralogous and homologous gene families often present in species that have gone through a whole genome duplication event. The contiguity of the short-read (454 + Illumina) only assembly showed a dramatic 40-fold improvement of the N50 length (from 1.3 kb to 51.5 kb) upon the addition of ~27× coverage long-read PacBio sequence data (Fig. 1 and Table 1). A substantial portion (~74%) of our 454/Illumina contigs could be assembled into larger contigs/scaffolds using PacBio raw reads. Over 90% of the contigs that remained unassembled after the DBG2OLC scaffolding step had repeat contents of at least 50%, rendering the task of placing them into the assembly nearly impossible.

A recently introduced Chicago method can capture DNA linkage in the range of several hundreds of kilobases and use the information from proximity ligation libraries to obtain a highly contiguous genome assembly^19^. Upon the addition of the long-ranged Chicago data, the length of the N50 scaffold and the largest scaffold roughly doubled in our final assembly, compared to the preliminary draft (from 51.5 kb to 96.8 kb; Table 1). Moreover, the proportion of the assembly contained within scaffolds larger than 500 kb was noticeably higher after the Chicago data has been incorporated through the HiRise scaffolding (Fig. 1). Tang *et al*.^30^ reported a 2.3-fold improvement in the N50 length of the Reyan7-37-97 final assembly, from 55 kb to 1.28 Mb, upon the incorporation of BAC-end sequence data (from 47,616 BAC clones) to their Illumina shotgun and mate-pair based assembly^30^. Although Tang *et al*.^30^ obtained the assembly with slightly higher contiguity than ours using the BAC-end sequencing strategy, our approach of combining long-read PacBio and long-range Chicago data to scaffold contigs assembled from 454/Illumina short reads was probably more cost-effective and less time-consuming as it did not involve the labour-intensive step of constructing BAC libraries.

To assess the quality of our *de novo* assembly, independent sources of publicly available rubber tree DNA and cDNA sequences were aligned to the assembled genome (Supplementary Table S2). These sequences included the first published draft genome assembly^13^, EST reads and transcriptome assembly obtained from 454 and Illumina sequencing^12^,^13^,^33^,^34^ and our PacBio Isoform Sequencing (Iso-seq) data. Over 99% of the scaffolds assembled by the Malaysian group could be aligned to our genome assembly. EST reads, Trinity-assembled transcripts from various tissues and full-length transcripts obtained from the Iso-seq showed mapping rates of 95.5%, 93.8% and 98.1%, respectively (Supplementary Table S2), indicating nearly complete coverage of the gene space in our assembly. We also investigated the coverage of the benchmarking universal single-copy orthologs (BUSCO)^35^ to assess the completeness of the gene space in the assembly. Of the 429 conserved eukaryotic genes, 403 (94%) were identified in the assembly, of which 337 (79%) were considered ‘complete’ (i.e., the length of the recovered genes are within two standard deviations of the BUSCO group mean length)^35^, suggesting that the genome assembly is relatively complete with respect to the gene space.

### Genetic anchoring of rubber tree whole genome assembly

Moving beyond fragmented genome assembly requires a high-quality and high-density linkage map that can help order and orient *de novo* assembled scaffolds into pseudo-chromosome scale sequences^22^. We employed a previously published high-density, genotyping-by-sequencing (GBS)-SNP based linkage map^11^ of BPM24 to anchor our genome assembly. Of the 2,321 markers present on the integrated genetic map, 2,308 SNPs were unambiguously located on the assembly and used to anchor and orient the scaffolds on 18 *H. brasiliensis* linkage groups. A total of 862 scaffolds spanning approximately 310 Mb (~24.7% of the assembled genome) were anchored to the linkage map, of which 418 scaffolds representing 198 Mb (64% of the anchored sequences) could also be oriented. When we combined our data with contig placement information available in the literature^13^, we were able to anchor 706 additional scaffolds covering 38.5 Mb onto the linkage map. These 1,568 anchored scaffolds, albeit representing only 28.9% of the genome assembly (363 Mb), were associated with over two thirds (30,024 out of 43,868) of the predicted protein-coding genes.

Since the genetic map used to anchor scaffolds was obtained from a GBS experiment that utilized methylation-sensitive enzymes to reduce genome complexity^11^, SNP markers identified were likely to be located in hypomethylated, non-repetitive regions. Consequently, the scaffolds that had been placed on the linkage map were associated predominantly with the euchromatic regions and encompassed a substantial portion (68%) of protein-coding sequences in the gene space. The percentage of the assembly assigned to the linkage map (~29%) appeared to represent the non-repetitive regions of the genome, which is congruent with previous and our findings that 69–72% of the rubber tree genome contained repetitive sequences^13^,^30^ (see ‘**Repeat content in the genome assembly’** below). Furthermore, repeat content analyses of anchored and unanchored scaffolds revealed that, on average, unanchored scaffolds contained ~80% repeat elements compared to 44.5% repetitive content of scaffolds that had been placed on the linkage map. High repeat content in the unanchored scaffolds rendered it virtually impossible to assign them specific location, especially when SNP markers derived primarily from hypomethylated euchromatic regions of the genome were used. The use of a linkage map constructed from a set of markers evenly distributed across the whole genome may be preferred under this circumstance. Similar situations in which the majority of genetically anchored scaffolds represented mainly the gene-rich portion of the genomes have been observed in watermelon^36^ and cucumber^37^.

### Repeat content in the genome assembly

RepeatModeler and RepeatMasker were used to identify and annotate the repeat elements in the genome assembly. These sequences represented 69.2% (869 Mb) of the assembled genome and were composed of 3.5 Mb (0.28%) of low complexity elements, 1.3 Mb (0.1%) of small RNA structures, 13 Mb (1.11%) of simple sequence repeats, 21 Mb (1.72%) of DNA transposons, 160 Mb (12.73%) of unclassified elements, and nearly 670 Mb (53.31%) of retrotransposons (Table 2). Similar percentages of repeat contents were also observed in cottons (upland cotton^38^, 67%; sea island cotton^39^, 69%), eggplant^40^ (70%), and bread wheat^41^ (76%). A significant portion of unknown classification suggests that rubber tree possesses unique repeat elements not represented in RepBase. Among the classified elements, retrotransposons were the largest group of mobile elements, occupying over half of the assembled genome. The majority of retrotransposons were classified as long terminal repeats (LTR; 51.55%), which could further be categorized into *Copia*-like (10.23%) and *Gypsy*-like (37.75%) elements, both of which were also the most abundant LTR subfamilies in other sequenced Euphorb genomes^42^,^43^,^44^ (Supplementary Fig. S2).

Rahman *et al*.^13^ and Tang *et al*.^30^ reported similar estimates of 71% and 72% repeat contents in the assemblies of RRIM600 and Reyan7-33-97 accessions, respectively. The percentage of *Gypsy* and *Copia* LTR elements was also comparable between this and previously published rubber tree genome assemblies^13^,^30^. Proportions of transposable elements in the four Euphorbiaceae species (rubber tree, cassava, castor bean and physic nut) varied from 37% to ~70%, with higher repeat contents in larger genomes. *Hevea* genome appeared to harbour a substantially greater portion of repetitive elements (69–72%) compared to the genomes of its close relatives (cassava^21^, 50%; castor bean^42^, 52%; physic nut^43^, 37%), suggesting a *Hevea*-specific expansion of transposable elements after speciation events. Intriguingly, among the classified elements, the proportion of LTRs present in rubber tree (51.5%) seemed to be higher than that present in cassava^21^ (32.5%), physic nut^43^ (29.9%) and castor bean^42^ (16.2%; Supplementary Fig. S2). There might be a period after the split of rubber tree and cassava lineages during which LTRs have undergone an extensive proliferation in rubber tree, and this could partly be responsible for the expansion of *H. brasiliensis* genome. Recent bursts of three LTR families in *Oryza australiensis*, a wild relative of the cultivated rice, have resulted in the accumulation of a large number of retrotransposon copies during the last three million years and were primarily responsible for a twofold increase in its genome size^45^. Besides the variation in percentages of LTRs present, the composition of LTRs also varied among euphorb genomes. The ratio of *Gypsy* to *Copia* elements ranged from 6.8:1 in cassava^21^, 3.7:1 in rubber tree, 2.5:1 in physic nut^43^ and 2.3:1 in castor bean^42^.

### Functional annotation of protein-coding sequences

Protein-coding sequences were annotated using information from multiple *ab initio* gene prediction tools, homology searches against sequence database and the transcript alignment program PASA. Sequencing of PacBio Iso-seq libraries generated 379,436 raw reads totalling 6.4 Gb. A total of 54,852 full-length transcripts (46,129 non-redundant transcripts) were identified based on the presence of 5′ and 3′ cDNA primers and a polyA tail signal preceding the 3′-primer. These unique full-length transcripts along with assembled unigenes from published RNA-seq data obtained from different tissues (leaf, bark, latex, root)^13^,^30^,^33^,^46^ were used as transcript evidences, which were subsequently combined with outputs from gene prediction programs and homology searches using the program EvidenceModeler (EVM) to obtain a consensus set of 45,236 predicted genes. After removing gene models whose coding sequences overlap with repeats or those that contain intervening stop codons, a total of 43,868 protein-coding loci were predicted. In rubber tree, the average gene length (2,747 bp), average exon length (223 bp), and average number of exons per gene (4) were comparable to those in cassava^44^ and castor bean^42^ (Supplementary Table S3). The GC contents of the coding sequences and the introns are 43.1% and 32.3%, similar to the previous estimates of 41.5% and 32.6%^30^, respectively (Supplementary Table S3).

**Figure 2:**
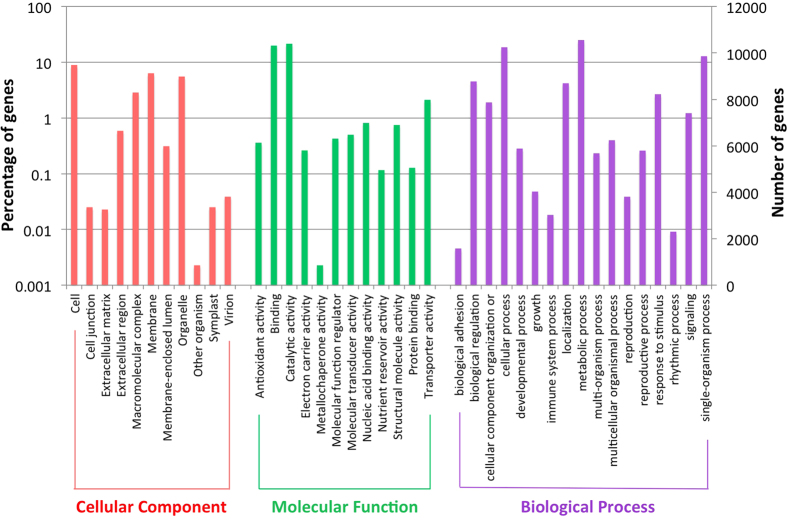
Gene Ontology (GO) annotation of rubber tree genes in the genome assembly. Results are summarized in three main categories: cellular components, molecular functions and biological processes. A total of 20,107 genes have been assigned GO terms.

Of the 43,868 predicted genes, 30,232 (68.92%) were assigned InterPro motifs and 33,718 (76.9%) and 16,794 (38.3%) had significant BLASTP matches to proteins in Genbank non-redundant protein and SwissProt databases, respectively. Predicted protein-coding genes were further assigned functions by aligning them to the best matches in the Genbank non-redundant protein database. Blast2Go suite used the BLASTP results to map and retrieve GO annotation for 20,107 sequences (Fig. 2). The most prevalent GO term associated with biological processes were metabolic process genes (10,983), followed by cellular process genes (8,153), single-organism process gene (5,631) and ten additional groups with 50 or more terms. Among genes annotated to various molecular functions, the largest category was catalytic activity (9,423), followed by binding activity (8,729) and another nine subcategories with 50 or more terms. Additionally, 44.1% of the predicted genes could be functionally annotated using KEGG database (Supplementary Fig. S3) and 31.43% could be assigned Enzyme Commission (EC) classification. The most abundant InterPro and Pfam domains were pentatricopeptide repeats followed by protein kinase and Leucine-rich repeat domains (Supplementary Fig. S4). In addition to protein-coding genes, we identified 623 transfer RNA (tRNA), 274 ribosomal RNA (rRNA), 282 small nucleolar RNA (snoRNA), 164 small nuclear RNA (snRNA) and 193 micro RNA (miRNA) genes in the BPM24 assembly (Supplementary Table S4). The statistics of non-coding RNAs discovered here resembled those previously reported for RRIM600^47^ and Reyan7-33-97^30^.

**Figure 3:**
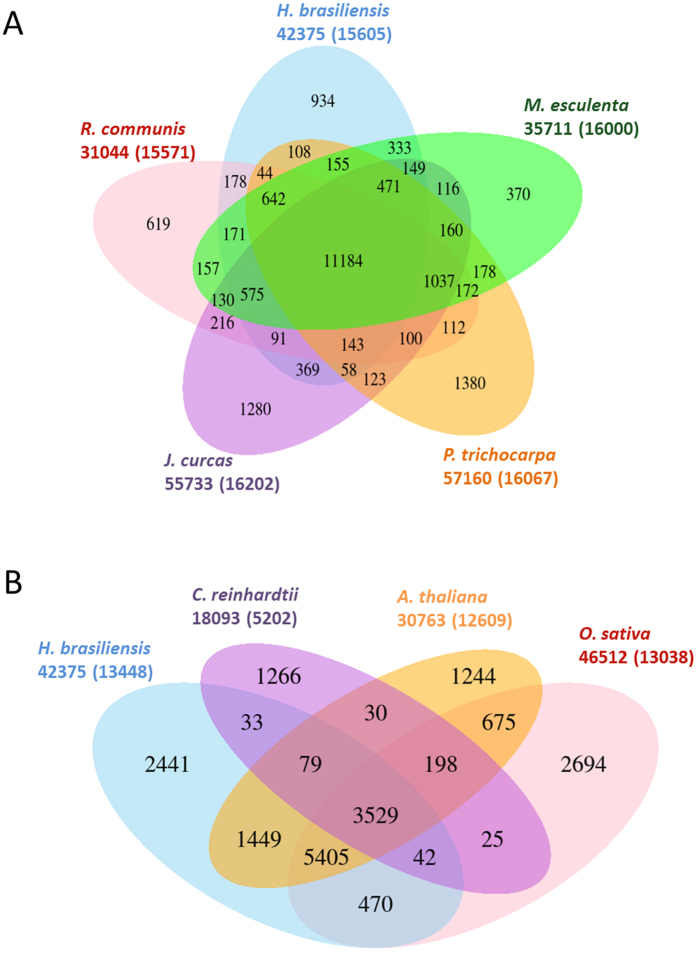
Venn diagrams displaying clusters of shared and unique orthologous gene families in (**A**) Euphorbiaceae family and (**B**) among representative species in plant lineage as identified by OrthoMCL. The number of genes in the families and the number of gene families are indicated under the species name in bold and parentheses, respectively.

We performed comparative analyses of the complete gene sets of rubber tree, other sequenced Euphorbiaceae species and black cottonwood using OrthoMCL. A total of 42,375 genes in the rubber tree genome were assigned into 15,605 orthologous gene clusters. Among these clusters, 11,184 appeared to be common to all five species and 11,759 were unique to Euphorbiaceae family (Fig. 3A). There were 934 gene families specific to rubber trees, which were significantly enriched with genes related to molecular function categories such as catalytic and binding activities. Among plant species with available genome sequences, cassava shares the most recent common ancestor to rubber tree, and consequently, they shared the largest number of orthologous gene clusters (13,680 families), representing 87.6% and 85.5% of total gene clusters in rubber tree and cassava, respectively. When compared with orthologous gene sets from rice (*O. sativa*), Arabidopsis and Chlamydomonas, rubber tree shared 3,529 clusters with representative species from monocotyledons, dicotyledons and green algae (ancestors of terrestrial plants), respectively (Fig. 3B).

### Disease resistance genes in rubber tree

Plants have evolved two branches of innate immunity to defend potential pathogens. The basal defence system uses pattern recognition receptors that respond to pathogen-associated molecular patterns (PAMPs) whereas the R-gene mediated defence relies on the recognition of pathogen-secreted effectors by the polymorphic NBS-LRR proteins^48^. A total of 1,275 putative pattern-recognition receptor (PRR) genes were identified in the rubber tree genome. Almost 90% of the PRR proteins in *Hevea* are receptor-like proteins (RLP) with the remaining being plasma membrane receptor-like kinases (RLK). The RLP family has expanded considerably in rubber tree (1,135) and cassava (1,169) - the size of RLP family in these two species have roughly doubled compared to castor bean (706), Arabidopsis (580) and monocot species such as rice (493) and maize (515; Supplementary Fig. S5). The apparent expansion of the RLP family may indicate that in evolution these receptors have become one of the preferred systems for non-self recognition in plants.

A large number of putative nucleotide-binding site (NBS) and leucine-rich repeat (LRR) containing R genes were identified in the rubber tree genome (Supplementary Fig. S5). The NBS-LRR proteins can be further subdivided based on the presence of N-terminal coiled-coil (CC) or toll/interleukin receptor (TIR) motifs; nevertheless, some predicted NBS-LRR genes have no obvious TIR or CC domain. Rubber tree appeared to have a significantly larger number of these predicted NBS-LRR proteins compared to CC-NBS-LRR and TIR-NBS-LRR proteins, similar to its close relatives cassava and castor bean (Supplementary Fig. S5). An opposite situation was observed in Arabidopsis where CC-NBS-LRR and TIR-NBS-LRR proteins both outnumbered the NBS-LRR. Compared to Arabidopsis, there seemed to be an expansion in the NBS-LRR gene families in euphorb species.

We calculated the substitution ratio of non-synonymous (*Ka*) to synonymous (*Ks*) mutations for different R-gene families, and the results showed that *Ka/Ks* ratios of RLK (0.42), RLP (0.47), and TIR-NBS-LRR (0.42) encoding genes were similar to the genome-wide average ratio of all paralogous groups (0.45). On the other hand, the average *Ka/Ks* value for CC-NBS-LRR gene families (0.61) was significantly higher than the genome-wide average (a two-tailed *t* test, *P-*value < 0.01), indicating greater functional constraint during the evolution of TIR gene subfamilies and stronger diversifying selection in CC-NBS-LRR gene families^49^, which could provide variation that would allow rubber tree to adapt to different pathogens. Putative NBS-LRR resistance genes that were anchored to the 18 linkage groups did not show a uniform distribution across the genome (Supplementary Fig. S6). Nearly half of those NBS genes appeared to be located in clusters, supporting the argument that they have evolved predominantly through tandem duplications, similar to what have been reported in other sequenced plant genomes^50^,^51^.

### Whole genome duplication and phylogenetic analyses

**Figure 4:**
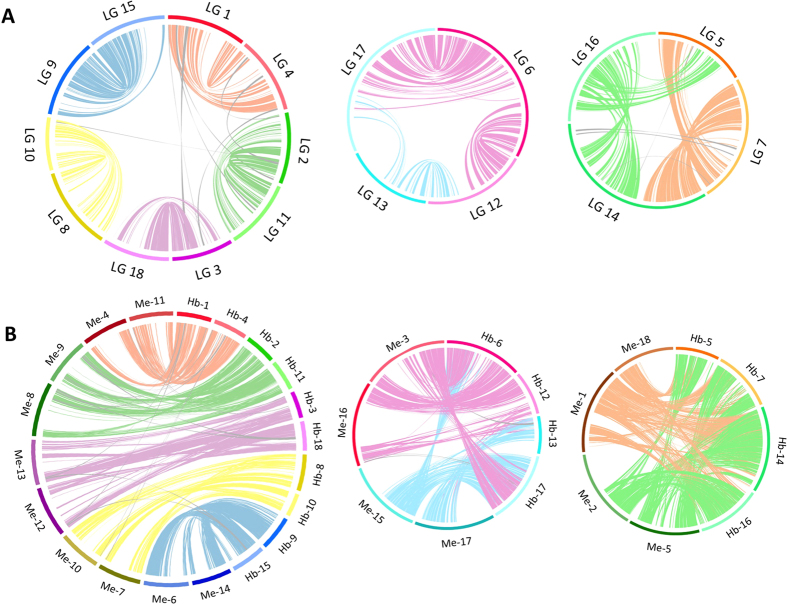
Syntenic analysis of 18 linkage groups suggests paleotetraploidy in *H. brasiliensis*. (**A**) Syntenic relationship of gene blocks among rubber tree linkage groups is illustrated with CIRCOS plots. A large circle displays 1:1 synteny between five duplicated pairs of linkage groups while two smaller circles indicate linkage groups that share syntenic regions with two other linkage groups. Lines are coloured by the paralogous regions. (**B**) Diagrams showing genetic colinearity between *H. brasiliensis* and *M. esculenta.* Lines link the position of orthologous gene sets with the line colour representing each linkage group/chromosome set. Rubber tree linkage groups are designated with “Hb” followed by the linkage group numbers, and cassava chromosomes are designated with “Me” followed by the chromosome numbers.

Whole genome duplication followed by gene loss and diploidization is common in most eudicots and has been recognized as a major evolutionary force that gives rise to neofunctionalization in plants. We scanned the rubber tree genome for intragenic homeologous segments on the basis of similarity in protein coding sequences. We defined those segments as runs of at least ten genes with colinear (or nearly colinear) runs of paralogs elsewhere in the genome with fewer than six intervening genes. A total of 164 syntenic blocks containing 2,951 paralogous gene pairs were identified in the rubber tree genome. Comparative analyses of these intragenomic homeologous regions clearly suggested that rubber tree has experienced a paleotetraploidization event (Fig. 4A). The distribution of paralogous gene pairs across the 18 linkage groups unambiguously revealed one-to-one synteny between five pairs of homeologous linkage groups that likely arose from a recent whole genome duplication event. We also uncovered more complex signals of duplications and rearrangements in two sets of four linkage groups (Fig. 4A). A similar pattern of conserved synteny between five pairs of chromosomes and two groups of four chromosomes has also been detected in cassava^21^. Figure 4B illustrates stretches of conserved syntenic genes between rubber tree and cassava. The degree of macrosynteny conservation observed between these two euphorbs clearly supports the hypothesis that the paleotetraploidy event took place prior to the divergence of the *Hevea* and *Manihot* species.

**Figure 5:**
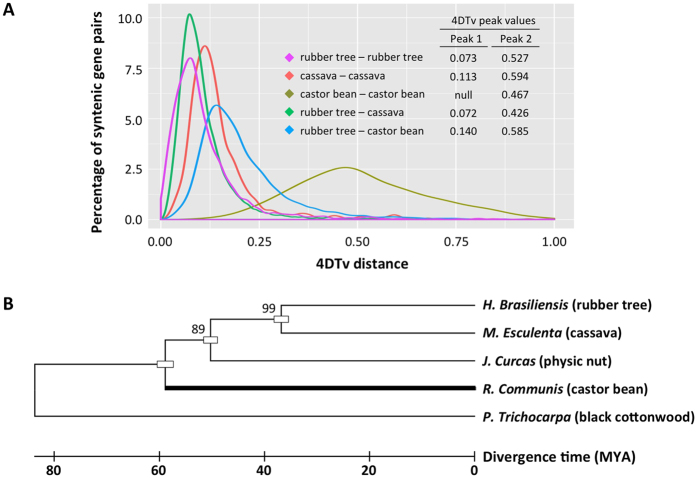
(**A**) Distribution of 4DTv distances between syntenic gene pairs among rubber tree, cassava and castor bean. Segments of homologous genes were identified by locating blocks of BLAST hits (E-value 1 × 10^−10^) containing at least ten syntenic genes with less than six intervening sequences between them. The 4DTv distances between these paralogous/orthologous genes from these segments are illustrated. (**B**) A maximum likelihood phylogenetic tree of Euphorbiaceae species, with *black cottonwood* as an outgroup, based on orthologs of single-gene families. The numbers at each node display bootstrap values. The divergence time was calibrated to 58 MYA (thick black bar) using fossil evidence from Magallon *et al*. (1999)^55^.

We estimated the relative ages of the paralogous gene pairs in rubber tree using the accumulated nucleotide divergence at fourfold synonymous third-codon transversion position (4DTv) values. A prominent peak in 4DTv values, corrected for multiple substitutions, was observed at 0.073 synonymous transversions per site, highlighting a recent *Hevea*-lineage specific paleotetraploidization (Fig. 5A). Most paleopolyploid plants have undergone diploidization through structural and functional alterations, including genomic reorganization, differential mutation, elimination and inversion in duplicated chromosomes, and eventually restored a diploid-like behaviour of the polyploid genomes^52^. Paralogous genes may evolve in several ways following the whole genome duplication. Due to functional redundancy, some of the duplicated genes may be lost or subjected to neofunctionalization while others may be degraded into non-functional genes^53^ or have diverged too much to be detected. Since the whole genome duplication occurred after the split of the castor bean and rubber tree lineages^21^, one would expect a gene in castor bean to have two homologs in rubber tree. A similarity search with BLASTP revealed that 12,677 protein sequences in castor bean were best hits of two of fewer predicted proteins in rubber tree. Of these, we identified two rubber tree homologs for 7,054 (55.6%), suggesting that both copies had been retained. For the remaining 5,623 proteins, we could detect only one rubber tree homolog, suggesting that the duplicate had either been lost or diverged beyond recognition.

To analyse the evolutionary divergence of rubber tree and other species, we used the transversion rate at fourfold degenerate sites to analyse orthologous gene pairs identified in syntenic blocks between rubber tree and its close relatives (Fig. 5A). The 4DTv values between paralogs in cassava peaked at 0.113, indicating a recent species-specific duplication. The identical chromosome numbers (*n* = 18) of rubber tree and cassava, which is almost twice as high as that of castor bean (*n* = 10), also supports the hypothesis that a shared whole genome duplication event took place before the speciation of *Hevea* and *Manihot*. The orthologs between rubber tree and cassava and rubber tree and castor bean showed 4DTv distance peaks at 0.072 and 0.14 respectively (Fig. 5A), consistent with a more ancient divergence between rubber tree and castor bean. The absence of the left sharp peak in castor bean appeared to indicate its lack of species-specific duplication. Only a broad peak on the right was observed at 0.467 synonymous transversions per site represented an ancient duplication shared among eudicots^54^ (Fig. 5A). To estimate speciation times, we constructed a phylogenetic tree based on orthologs of single-gene families in rubber tree and other euphorb species, with black cottonwood as an outgroup (Fig. 5B). A fossil-calibrated molecular clock for the Euphorbiaceae placed the origin of the family at 58 million years ago (MYA)^55^. The maximum likelihood tree suggested a speciation time of around 36 MYA for *Hevea* and *Manihot*, and a divergence time of *Ricinus* lineage from other euphorbs around 60 MYA, agreeing with the estimates from previous phylogenetic analyses^21^.

### Iso-seq and alternative splicing

**Figure 6:**
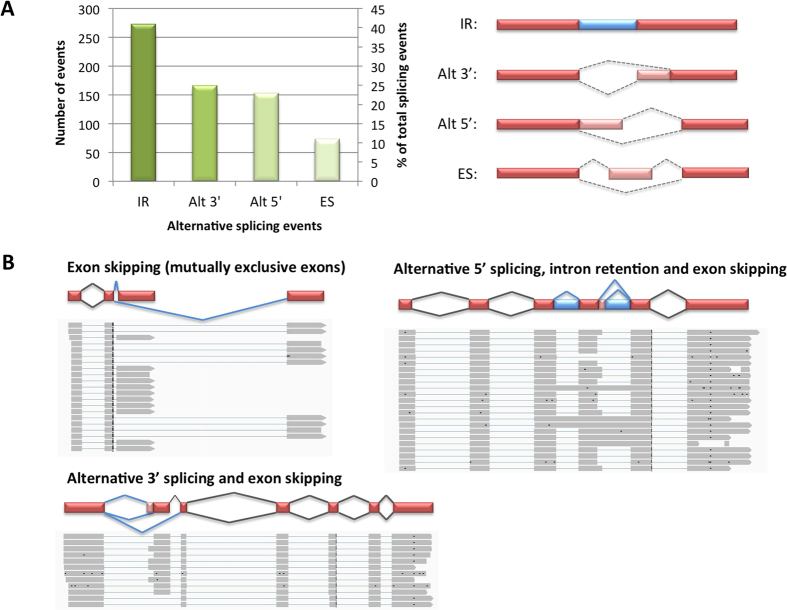
(**A**) Distribution of alternative splicing events in rubber tree, with visualization of each mode. Alt 3′, alternative 3′ acceptor site selection; Alt 5′, alternative 5′ donor site selection; ES, exon skipping; IR, intron retention. (**B**) Examples of genes that display different types of alternative splicing. Drawings of gene models showing possible splicing events (indicated by blue lines) are shown at the top and aligned PacBio Iso-seq reads are shown at the bottom.

The third generation sequencing platform has provided us a unique opportunity to investigate alternative splicing with its ability to obtain full-length cDNAs, including long transcript isoforms. PacBio allows us to bypass a time-consuming and labour-intensive step of cloning individual full-length cDNAs and sequencing them using traditional Sanger approach. It also eliminates the need to assemble transcriptome data obtained from short-read platforms as PacBio technology is capable of generating long single-molecule reads of up to 20 kb, providing direct evidence for alternative transcript isoforms. Besides the analysis of alternatively spliced *HbJAZ* transcripts^56^, there has not been a report on alternative splicing in rubber tree. We used our PacBio Iso-seq data to identify full-length transcripts and study their isoforms. We first clustered 54,852 reads with poly(A) sites into 11,784 groups with a minimum depth of 5 reads/group and used SpliceGrapher^57^ to identify transcript variants. Of those, 1,481 groups were from intronless genes and the remaining 10,303 were derived from genes that contained at least one intron. We detected a total of 636 alternative splicing events from intron-containing genes, with the majority of events (41%) being intron retention, followed by alternative 3′ splicing (25%), alternative 5′ splicing (23%) and exon skipping (11%; Fig. 6A), similar to the distributions of alternative splicing modes observed in maize^58^ and sorghum^59^. Relative prevalence of each type of alternative splicing appears to vary between different taxa. While the predominant mode of alternative splicing in metazoans is exon skipping and the least common form is intron retention^60^,^61^,^62^, plant species including rubber tree use intron retention as the primary form, and exon skipping only accounts for a small portion of alternative splicing^63^,^64^. Different types of alternative splicing can occur in a combinatorial manner, and a single gene may be subjected to more than one alternative splicing mode. Examples of transcripts with different types of alternative splicing are shown in Fig. 6B. Tissue-specific isoforms are well recognized in plants and several studies have shown that responses to abiotic stress significantly impact alternative splicing of pre-mRNAs^63^,^65^. Since our Iso-seq data were obtained from young leaf tissue under normal growth condition, the number of alternative splicing events above is likely to be underestimated. Nevertheless, this preliminary analysis provided us the first glimpse of an alternative splicing overview in rubber tree. Further analyses using RNA samples from various tissues and different growth conditions (biotic and abiotic stresses) will be required to thoroughly probe the complete repertoire of transcript isoforms present in this species.

In conclusion, a 1.26-Gb *de novo* assembly of the BPM24 rubber tree genome presented here serves as an example that sequencing a highly heterozygous genome is feasible by combining 454/Illumina short-read and PacBio long-read data. We demonstrated the significance of utilizing long PacBio reads to resolve ambiguous regions containing primarily repetitive sequences. Since PacBio raw reads suffer from a high error rate, it may be costly to collect these long reads to sufficient depth to span most of the large repeats. We presented an efficient and affordable approach that employed both long-read PacBio and long-range Chicago data to scaffold contigs assembled from deep-coverage short reads and demonstrated that both techniques contributed significantly to remarkable improvements in sequence contiguity. Although there have been reports on the anchoring of scaffold sequences on linkage maps in rubber tree^11^,^13^,^66^, our attempt to place and orient the assembled genome yielded a considerably larger set of genetically anchored sequences (covering 363 Mb). This allowed us to investigate whole genome duplication events and perform comparative genomic analyses in *H. brasiliensis* for the first time. The distribution of paralogous gene pairs across the linkage groups clearly revealed that rubber tree genome has experienced a paleotetraploidization event. Moreover, the stretches of conserved syntenic genes between rubber tree and cassava supports the hypothesis that the paleotetraploidy event occurred prior to the *Hevea-Manihot* speciation. The genome assembly reported here is a valuable resource for studying marker-trait association at a whole genome level and for developing desirable high-yielding cultivars to meet growing demands for natural rubber.

## Methods

### Plant materials, sample preparation and sequencing

Leaf tissues were collected from the apical shoots of rubber tree clone BPM24 maintained at the Rubber Research Institute of Thailand, Ministry of Agriculture and Cooperatives (Thailand). Fresh tissue samples were collected, immediately frozen in liquid nitrogen and stored at −80 °C until use. For whole genome shotgun sequencing, frozen leaf samples were pulverized in liquid nitrogen, and DNA was isolated using a DNeasy Plant Mini Kit (Qiagen, Valencia, USA). DNA quantity was measured using a NanoDrop ND-1000 Spectrophotometer, and prior to sequencing, its integrity was verified by a Bioanalyzer (Agilent Technologies, Santa Clara, USA). Whole genome sequencing was performed using 454 GS-FLX+ (Roche Applied Science, Indianapolis, USA), Illumina HiSeq 2000 (Illumina, San Diego, USA) and PacBio RS II (Pacific Biosciences, Menlo Park, USA) systems, according to each manufacturer’s instruction. A total of ten sequencing runs were performed with ~600 bp insert single-end library and two sequencing runs were carried out for paired-end libraries (with insert sizes of 8 kb and 20 kb) on the 454 GS-FLX+ platform. One Illumina paired-end library (2 × 100 bp) was prepared and sequenced on the HiSeq 2000 by Macrogen (Seoul, Korea). Three libraries with the insert sizes of approximately 10,000 bp were constructed for the PacBio RS II sequencing system. A total of 90 cells were sequenced using the P6-C4 polymerase and chemistry with 240 min movie times.

### Proximity ligation (Chicago) library preparation and sequencing

Long-ranged mate pairs were produced using the Chicago method and sequenced by Dovetail Genomics (Santa Cruz, USA). DNA for Chicago libraries was extracted from dark-adapted, young leaf tissue yielding >900 μg of high molecular weight (HMW) genomic DNA. Six Chicago libraries were prepared as described previously^19^. Briefly, 500 ng of HMW genomic DNA was reconstituted into chromatin *in vitro* and fixed with formaldehyde. Fixed chromatin was digested with *Mbo*I; the 5′ overhangs were filled in with biotinylated nucleotides, and free blunt ends were ligated. After ligation, crosslinks were reversed and the DNA purified from protein. The DNA was sheared to ~350 bp mean fragment size, and sequencing libraries were generated using NEBNext Ultra enzymes (New England Biolabs, Ipswich, USA) and Illumina-compatible adapters. The libraries were sequenced on an Illumina HiSeq 3000/4000 to produce 2 × 150 bp read pairs of insert sizes between 1 to 50 kb.

### RNA isolation, PacBio Iso-Seq library preparation and sequencing

Total RNA was extracted from leaf tissues using the Concert Plant RNA Reagent (Invitrogen, Carlsbad, USA). Poly(A) mRNAs were enriched from total RNA samples using the Absolutely mRNA Purification Kit (Agilent Technologies, Santa Clara, USA). The RNA integrity was assessed with a Bioanalyzer (Agilent Technologies, Santa Clara, USA) prior to the construction of the Iso-Seq library. We prepared the Iso-Seq library according to Pacific Biosciences’ Iso-Seq protocol using the SMARTer PCR cDNA Synthesis Kit (Clontech, Mountain View, USA) and the BluePippin Size Selection System (Sage Science, Beverly, USA). cDNA libraries were selected for the following bins: 1–2 kb, 2–3 kb, and 3–6 kb and sequenced on a PacBio RS II instrument using P4-C6 polymerase and chemistry with 240 min movie times.

### Genome Assembly

#### 454, Illumina, PacBio assembly

Illumina raw reads were filtered to obtain high quality sequence data by removing adapter-only reads, reads containing more than 5% unknown nucleotides and low-quality reads comprising more than 50% bases with Q-value of ≤20. Clean reads were assembled into contigs using SOAPdenovo v1.05^27^ and clustered using CD-HIT^28^ with minimum similarity cutoff of 95%. High quality, adapter-trimmed 454 reads were assembled together with Illumina consensus contigs using Newbler 2.8 (Roche Applied Science, Indianapolis, USA). The Newbler software supports FASTA/FASTQ input along with native 454 reads. To avoid the assembly failure in Newbler due to long FASTA sequences, the consensus Illumina input sequences longer than 2 kb were processed into 1.5 kb pseudo-reads with 300 bp overlap using the fb_dice.pl script from the FragBlast module (http://www.clarkfrancis.com/codes/fb_dice.pl). The assembled contigs and raw PacBio reads were used as inputs for DBG2OLC assembler^29^. We chose a DBG2OLC method because of its speed and its ability to perform the assembly using less than 30× coverage of long read molecules. Parameters used for DBG2OLC were as follows: k – 17, KmerCovTh – 5, MinOverlap – 30, AdaptiveTh – 0.01 and RemoveChimera – 1. The preliminary assembly obtained from 454, Illumina and PacBio sequencing data was subsequently used as an input for a long-range scaffolding approach.

#### Scaffolding the draft genome with HiRise

The preliminary assembly (1,249.5 Mb with a scaffold N50 of 51 kb), Illumina shotgun sequence data, and Chicago library read pairs in FASTQ format were used as input data for HiRise, a software pipeline designed specifically for using Chicago data to assemble genomes^19^. Shotgun and Chicago library sequences were aligned to the draft input assembly using a modified SNAP read mapper (https://github.com/robertDT/dt-snap). The parameters used for Chicago data are as follows: -ku -as -C + -tj GATCGATC -mrl 20, where “-as” specified that each read in the read pair should be aligned separately (i.e., no assumption about read pair separation or relative orientation) and “-tj GATCGATC” specified that any sequence seen after the Chicago junction sequence (GATCGATC) should be ignored for the purpose of aligning the reads. For shotgun data, the same parameters were used except for the “-as” and “-tj GATCGATC” options. The HiRise software also masked out four different types of regions in the input assembly: gaps (stretches of Ns), promiscuous regions (regions with many Chicago read pairs linking to map other input scaffolds), regions of high shotgun depth (regions with high depth of mapped shotgun reads relative to the rest of the assembly) and read deserts (regions of at least 1 kb with no Chicago reads mapping with map quality 20 or higher). The separations of Chicago read pairs mapped within draft scaffolds were analysed by HiRise to produce a likelihood model for genomic distance between read pairs, and the model was used to identify putative misjoins and score prospective joins. After scaffolding, shotgun sequences were used to close gaps between contigs.

### Assembly quality assessment and genetic map anchoring

Quality of genome assembly was evaluated by aligning available ESTs and Trinity-assembled transcript sequences using BLASTN at an E-value cutoff of 1 × 10^−10^ and an identity threshold of 90%. Completeness of the final assembly was assessed using Benchmarking Universal Single-Copy Orthologs (BUSCO)^35^. The OrthoDB BUSCO pipeline version 1.22 (released July 2016) was used to test for the presence and completeness of orthologs using the dataset for eukaryotes.

Scaffolds were anchored to the integrated genetic linkage map^11^ using 300 bp of SNP-flanking sequences extracted from the first published draft of the rubber tree genome^13^. Additional contigs/scaffolds were anchored by incorporating available scaffold data from the literature^66^. Scaffolds were subsequently ordered and those containing more than one SNP with a distinct genetic position were oriented.

### *De novo* repeat finding and repeat masking

RepeatModeler version 1.0.8 (http://www.repeatmasker.org/RepeatModeler.html) was run on the unannotated assembly to identify and classify *de novo* repeat families. RepeatModeler employs two *de novo* repeat-finding programs, RECON version 1.08 and RepeatScout version 1.0.5, to identify repeat element boundaries and to build consensus models of putative interspersed repeats. Alignment to Genbank’s non-redundant protein database (using BLASTX with an E-value cutoff of 1 × 10^−6^) was carried out to ensure that repeat sequences in the library did not contain large families of protein-coding genes that are not transposable elements. Repeat masking was performed on the assembled genome by RepeatMasker version 4.0.6 (http://www.repeatmasker.org/) against the repetitive sequences in RepeastMasker consensus library (20150807; www.girinst.org) and the custom species-specific repeat library generated by RepeatModeler.

### Gene prediction and annotation

Gene prediction and protein-coding sequence identification were conducted through a combination of transcriptome-based prediction, homology-based prediction and *ab initio* prediction methods using EvidenceModeler (EVM) version 1.1.1 r2015-07-03^67^. Transcriptome-based prediction methods combined information from PacBio Iso-seq and other available transcriptome databases. Iso-seq raw reads containing both 5′ and 3′ adapters (derived from full-length transcripts) were identified, and the adapters and poly(A) sequences were trimmed. Cleaned consensus reads were then mapped using Genomic Mapping and Alignment Program (GMAP; version r20160630) to the genome assembly^68^. In addition, PlantGDB-assembled unique transcripts (PUTs) obtained from *Manihot esculenta* (cassava), *H. brasiliensis, Ricinus communis* (castor bean), *Jatropha curcas* (physic nut), *Populus trichocarpa* (black cottonwood), and *Arabidopsis thaliana* were aligned to the genome with GMAP. Representative rubber tree transcriptome data from various tissues (apical meristem, leaf, bark, latex, root) obtained from public database (EST deposited in Genbank) and previously published reports^13^,^30^,^33^,^46^ were mapped to the genome assembly using PASA2 version 2.0.1^67^. *Hevea* protein sequences obtained from UniProt database were aligned to the unmasked genome using AAT version 1.52^69^. Three different *ab initio* gene predictors were run on the masked assembly. Protein-coding gene predictions were obtained with Augustus version 3.2.1^70^ trained with *H. brasiliensis* PASA transcriptome alignment assembly, GlimmerHMM version 3.0.3^71^ trained with *A. thaliana* and Geneid version 1.4.4^72^ trained with *Cucumis melo.* The parameters used for GlimmerHMM were as follows: acceptor_threshold – −8.47, donor_threshold – −3.45, ATG_threshold – −5.82, stop_threshold – −3.19. All gene predictions were combined by EVM to generate consensus gene models using the following weight for each evidence type: AAT – 0.2, PASA – 1, GMAP (IsoSeq) – 0.5, Augustus – 0.5, Geneid – 0.2 and GlimmerHMM – 0.3.

Gene functions were assigned according to best hit in a similarity search against NCBI non-redundant protein database using BLASTP with an E-value cutoff of 1 × 10^−5^. BLASTP results were imported into Blast2Go suite (version 2.8) for mapping and retrieving gene ontology (GO) terms associated with predicted sequences. The software also assigned the enzyme commission (EC) number and Kyoto encyclopedia of genes and genomes (KEGG) pathway annotations to the query sequences. Eukaryotic orthologous groups (KOG) assignment was extracted from BLASTP hits of STRING version 9.1^73^.

### Non-coding RNA annotation

tRNA genes were identified using tRNAscan-SE version 1.23^74^ using the covariance model TRNA2.cm with the tRNAscan parameters set to “strict” and EufindtRNA parameters set to “relaxed” with Int Cutoff of −32.1. rRNA genes were detected by aligning known plant rRNA sequences to the assembly using BLASTN with an E-value cutoff of 1 × 10^−5^. Other non-coding RNA, including microRNA (miRNA), small nucleolar RNA (snoRNA) and small nuclear RNA (snRNA) were identified using Infernal version 1.1.2^75^ using the Rfam 12.1 database^76^.

### Whole genome duplication and phylogenetic analysis

We aligned all rubber tree peptides against themselves using BLASTP (E-value cutoff of 1 × 10^−10^) to identify putative paralogs and the alignment results were subjected to the search for colinearity blocks using MCScanX^77^. Regions with at least ten syntenic genes (with the maximum of six intervening genes allowed) were used to define intragenomic homeologous regions. These regions were plotted using the software CIRCOS^78^. Protein-coding sequences from *M. esculenta*^21^, *R. communis*^42^, *J. curcas*^43^ and *P. trichocarpa*^79^ were downloaded and pairwise comparison between all input protein sequences was performed using BLASTP with an E-value cutoff of 1 × 10^−5^. Clustering was carried out based on a Markov cluster algorithm (MCL) using OrthoMCL^80^ software (version 2.0.9) with default parameters. Gene family numbers in each cluster were counted using a custom Perl script. Syntenic blocks between rubber tree and cassava or castor bean (at least ten syntenic genes and a maximum of six intervening genes allowed) were identified by MCScanX^77^ and plotted with CIRCOS^78^. The transversion of fourfold degenerate synonymous sites (4DTv) were calculated for each gene pair from the aligned blocks using the in-house perl script. A set of single-copy orthologs with representation in rubber tree, cassava, castor bean, physic nut and black cottonwood were selected for the construction of a maximum likelihood tree using MUSCLE^81^ software (version 3.8.31) for the multiple sequence alignment and MEGA5^82^ for phylogenetic tree construction. Bootstrapping was performed using 1000 repetitive samplings for each gene family. The *Ka/Ks* values were calculated using the ParaAT^83^ and KaKs_Calculator2.0 toolkit^84^.

## Additional Information

**Accession codes:** The *H. brasiliensis* (BPM24) genome assembly and Iso-seq sequences have been deposited in DDBJ/EMBL/Genbank databases under accession numbers BDHL01000001-BDHL01592579 (592579 entries) and DRA005328, respectively. Illumina, 454 and PacBio raw data are available at http://www4a.biotec.or.th/rubber/GenomeSeq.

**How to cite this article:** Pootakham, W. *et al*. *De novo* hybrid assembly of the rubber tree genome reveals evidence of paleotetraploidy in *Hevea* species. *Sci. Rep.* **7**, 41457; doi: 10.1038/srep41457 (2017).

**Publisher's note:** Springer Nature remains neutral with regard to jurisdictional claims in published maps and institutional affiliations.

## Supplementary Material

Supplementary Information

## Figures and Tables

**Table 1:** Rubber tree BPM24 assembly statistics.

	Short-read assembly 454 + Illumina contigs	Preliminary draft 454 + Illumina + PacBio scaffolding	Final assembly 454 + Illumina + PacBio & Chicago scaffolding
Number of contigs/scaffolds	989,097	658,583	592,580
Number of contigs/scaffolds > 1 kb	253,689	113,021	47,154
Total length of contigs/scaffolds (bp)	868,915,424	1,249,467,938	1,256,269,486
Longest contig/scaffold (bp)	76,143	1,224,126	2,026,921
Mean contig/scaffold size (bp)	878	1,897	2,119
Contig/scaffold N50 (bp)	1,316	51,412	96,825
Contig/scaffold L50	161,440	4,695	2,520
% N in scaffolds	0.002	0.00321	0.05463
Number of gaps	13,567	13,066	81,022
Non-gap bases	868,901,484	1,249,454,514	1,249,459,262

**Table 2:** Classification of repetitive sequences in the *H. brasiliensis* genome assembly.

Repeat element	Number of loci	Length occupied (bp)	Percentage of the assembly
DNA transposons	58,210	21,004,842	1.67
Retrotransposons:			
LINE	43,421	21,703,411	1.73
SINE	1,402	404,943	0.03
LTR: *Gypsy*	792,203	474271360	37.75
LTR: *Copia*	194,673	128587832	10.24
LTR: others	99,248	44,783,869	3.56
Simple repeats	289782	13,970,828	1.11
Low complexity	62563	3,522,906	0.28
Unclassified	578157	159,991,742	12.74
