# Supplementary material for: De novo hybrid assembly of the rubber tree genome reveals evidence of paleotetraploidy in Hevea species
Source: Sci Rep. 2017 Feb 2;7:41457. doi: 10.1038/srep41457 (PMC5288721; doi:10.1038/srep41457)
Supplement: Supplementary Information [file srep41457-s1.pdf]

Supplementary Information

***De novo* hybrid assembly of the rubber tree genome reveals evidence of paleotetraploidy in *Hevea* species**

Wirulda Pootakham[1], Chutima Sonthirod[1], Chaiwat Naktang[1], Panthita Ruang-Areerate[1], Thippawan Yoocha[1], Duangjai Sangsrakru[1], Kanikar Theerawattanasuk[2], Ratchanee Rattanawong[2], Napawan Lekawipat[2] and Sithichoke Tangphatsornruang[1,*].

[1]National Center for Genetic Engineering and Biotechnology (BIOTEC), National Science and Technology Development Agency, Pathum Thani, Thailand
[2]Rubber Authority of Thailand, Bang Khun Non, Bangkok, Thailand

Supplementary Table S1. Summary of rubber tree genome sequencing data

Sequencing platform	Number of reads	Number of bases	Genome coverage
**454 GS-FLX+:**	14,238,099	7,131,721,680	3.4×
1000-bp library	10,925,962	6,024,553,332	
8-kb library	1,368,995	536,328,992	
20-kb library	1,943,142	570,839,356	
**Illumina HiSeq 2000**	785,539,628	79,339,502,428	37.8×
**PacBio RSII**	6,434,943	56,308,060,886	26.8×

Supplementary Table S2. Assessment of genome assembly by comparing to publicly available genome and transcriptome data

Data source	Number of sequences	Number of sequences mapped	% of sequences mapped
EST (NCBI database)	51,701	49,355	95.46
Rahman et al. (2013) (NCBI accession numbers JT914190-JT981478)	67,289	65,367	97.14
Xia et al. (2011) (NCBI accession numbers GSE26514)	48,768	43,839	89.89
Li et al. (2012) (NCBI accession numbers JR344291-JR366936)	22,646	19,759	87.25
Triwitayakorn et al. (2011)	28,398	27,739	97.68
This study (PacBio Iso-seq)	45,968	45,093	98.10

Supplementary Table S3. Statistics for gene annotation

**Gene number**	43,868
**Total gene length (Mb)**	120.51
**Gene density**	9.59%
**Total exon number**	177,614
**Average number of exons/gene**	4.05
**Total exon length (Mb)**	39
**Average exon length (bp)**	222.5
**Minimum exon length (bp)**	2
**Maximum exon length (kb)**	6
**GC content of exons**	43.11%
**Total intron number**	133,746
**Average number of introns/gene**	3.05
**Total intron length (Mb)**	81.3
**Average intron length (bp)**	605.9
**Minimum intron length (bp)**	21
**Maximum intron length (kb)**	74.7
**GC content of introns**	32.31%

Supplementary Table S4. Non-coding RNA species identified in rubber tree whole genome assembly

**Class of RNA**	**Number**	**Mean length (bp)**	**Total length (bp)**
rRNA	274	494	135618
miRNA	193	130	25135
snoRNA	282	105	30161
tRNA	623	73	45776
snRNA	164	128	21006
Other ncRNA	129	135	17389

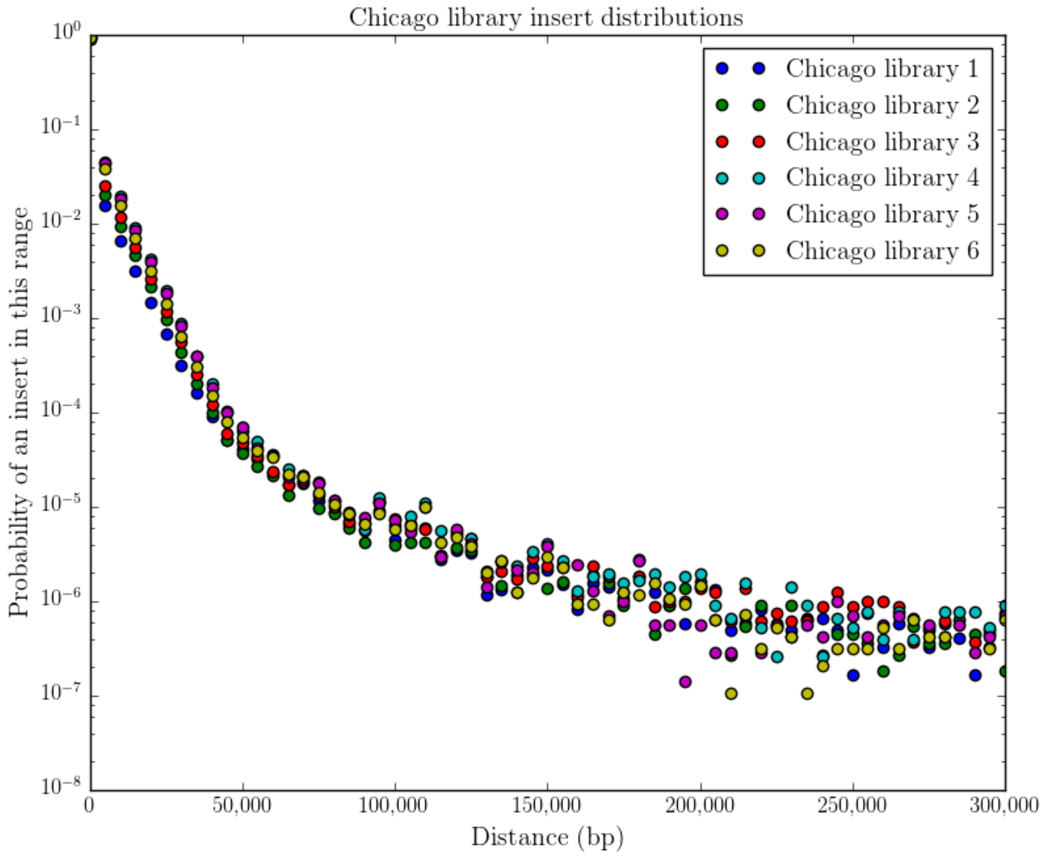

Supplementary Figure S1. Distribution of insert sizes in the Chicago libraries. The distance between the forward and reverse reads is given on the X axis in base pairs, and the probability of observing a read pair with a given insert size is shown on the Y axis.

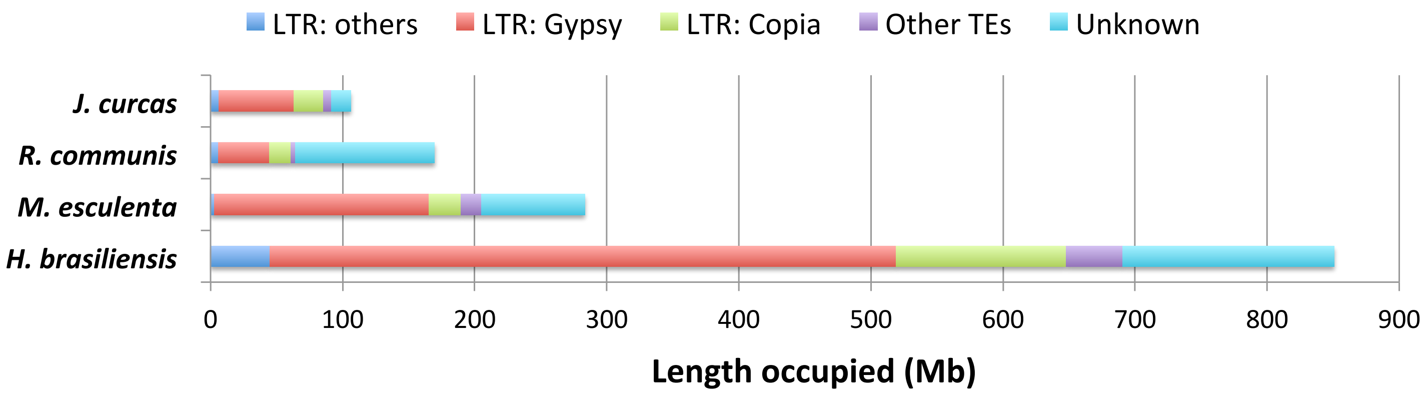

Supplementary Figure S2. Composition of repetitive elements in Euphorb genomes.

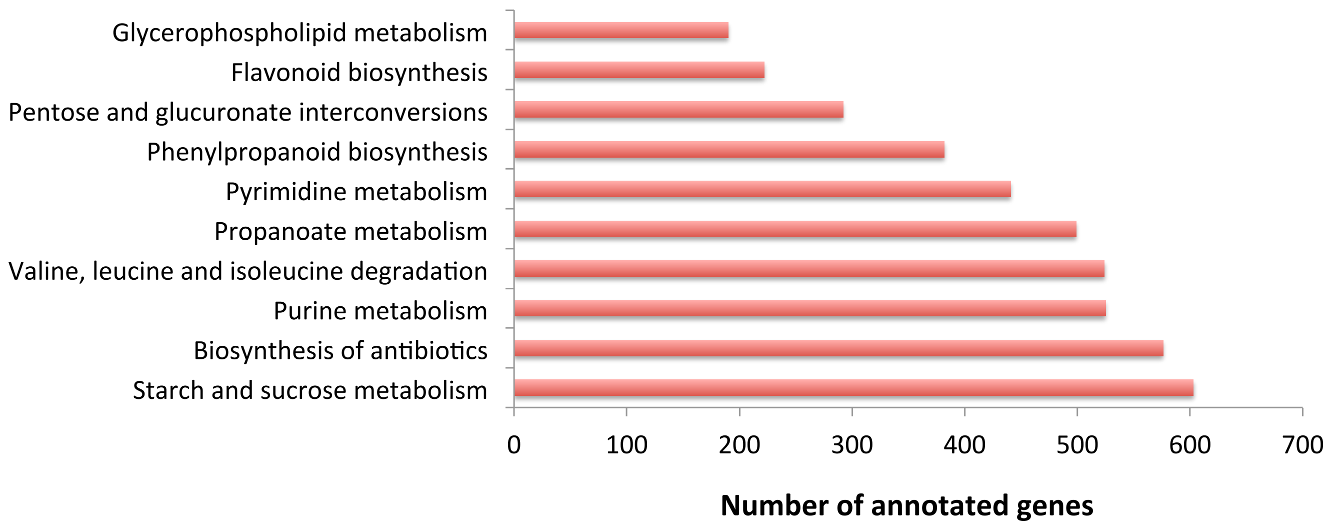

Supplementary Figure S3. KEGG metabolism pathway distribution for *H. brasiliensis* annotated genes.

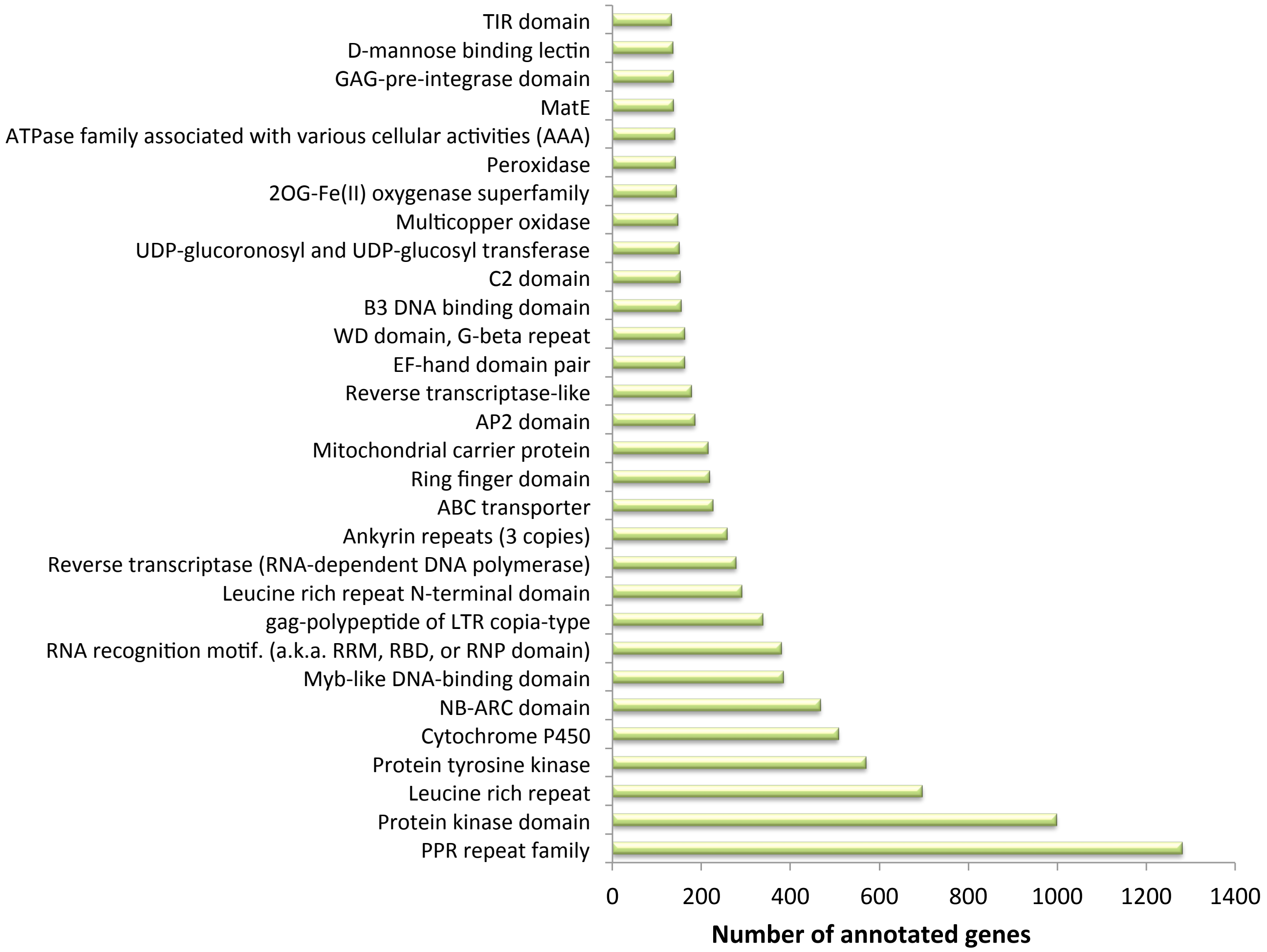

Supplementary Figure S4. Distribution of the top 30 Pfam domains identified in the rubber tree transcriptome.

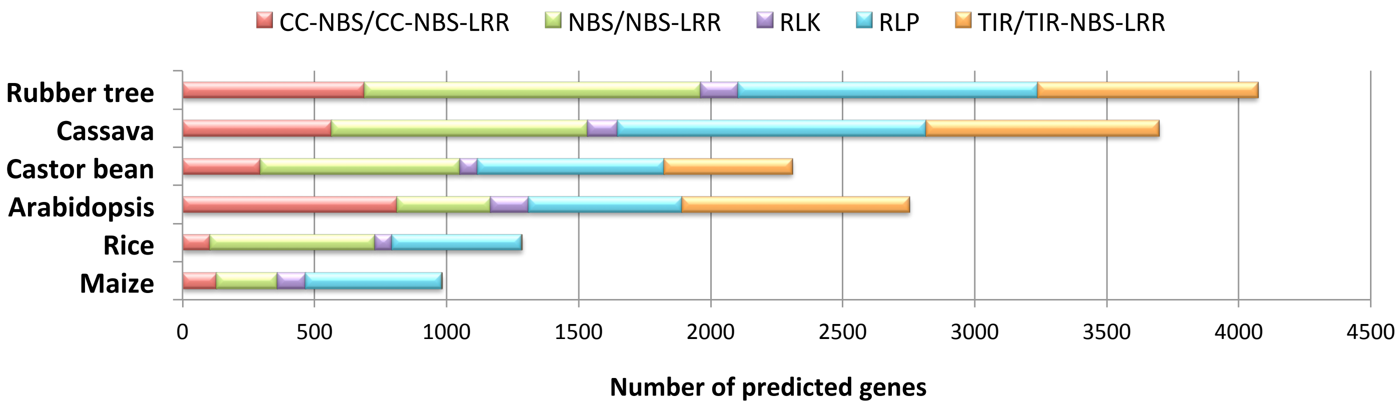

Supplementary Figure S5. Distribution of putative R-genes in rubber tree, cassava, castor beans, Arabidopsis, rice and maize identified using the Plant Resistance Gene database (PRGdb).

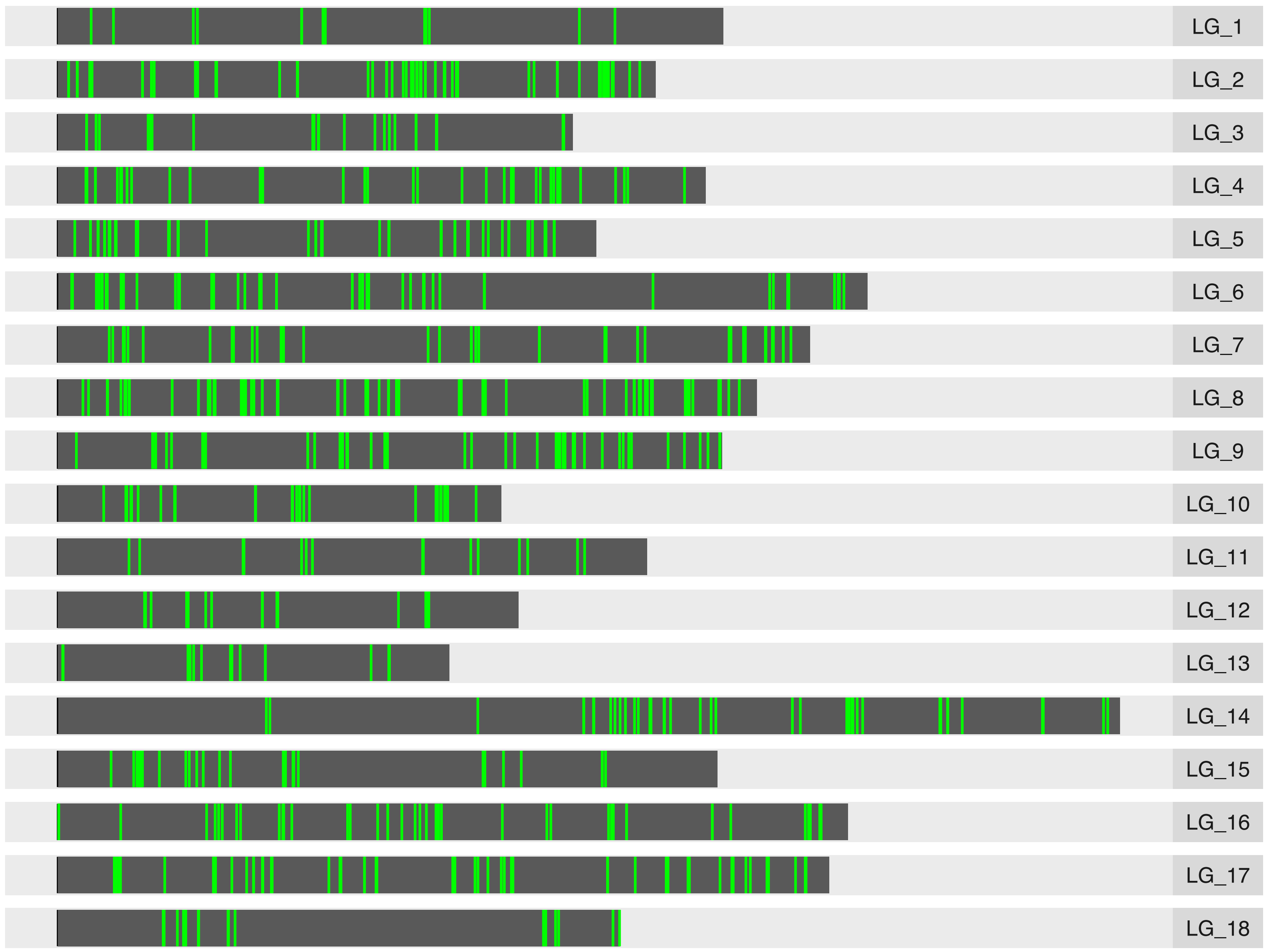

Supplementary Figure S6. Distribution of predicted NBS-LRR resistance genes in rubber tree. The relative genetic map position of each putative R gene is shown on the linkage map.
